# The role of intergenerational educational mobility and household wealth in adult obesity: Evidence from Wave 2 of the World Health Organization’s Study on global AGEing and adult health

**DOI:** 10.1371/journal.pone.0208491

**Published:** 2019-01-09

**Authors:** Stella T. Lartey, Costan G. Magnussen, Lei Si, Barbara de Graaff, Richard Berko Biritwum, George Mensah, Alfred Yawson, Nadia Minicuci, Paul Kowal, Godfred O. Boateng, Andrew J. Palmer

**Affiliations:** 1 Menzies Institute for Medical Research, University of Tasmania, Hobart, Australia; 2 Research Centre of Applied and Preventive Cardiovascular Medicine, University of Turku, Turku, Finland; 3 The George Institute for Global Health, University of New South Wales, Kensington, NSW, Australia; 4 Department of Community Health, University of Ghana, Accra, Ghana; 5 National Research Council, Neuroscience Institute, Padova, Italy; 6 World Health Organization (WHO), Geneva, Switzerland; 7 University of Newcastle Research Centre for Generational Health and Ageing, Newcastle, New South Wales, Australia; 8 Department of Nutrition, Harvard T.H. Chan School of Public Health, Boston, MA, United States of America; University of Kwazulu-Natal, SOUTH AFRICA

## Abstract

**Background:**

Obesity has emerged as a major risk factor for non-communicable diseases in low and middle-income countries but may not follow typical socioeconomic status (SES)-related gradients seen in higher income countries. This study examines the associations between current and lifetime markers of SES and BMI categories (underweight, normal weight, overweight, obese) and central adiposity in Ghanaian adults.

**Methods:**

Data from 4,464 adults (2,610 women) who participated in the World Health Organization’s Study on global AGEing and adult health (SAGE) Wave 2 were examined. Multilevel multinomial and binomial logistic regression models were used to examine associations. SES markers included parental education, individual education, intergenerational educational mobility and household wealth. Intergenerational educational mobility was classified: stable-low (low parental and low individual education), stable-high (high parental and high individual education), upwardly (low parental and high individual education), or downwardly mobile (high parental and low individual education).

**Results:**

The prevalence of obesity (12.9%) exceeded the prevalence of underweight (7.2%) in the population. High parental and individual education were significantly associated with higher odds of obesity and central adiposity in women. Compared to the stable low pattern, stable high (obesity: OR = 3.15; 95% CI: 1.96, 5.05; central adiposity: OR = 1.75; 95% CI: 1.03, 2.98) and upwardly (obesity: OR = 1.71; 95% CI: 11.13, 2.60; central adiposity: OR = 1.60; 95% CI: 1.08, 2.37) mobile education patterns were associated with higher odds of obesity and central adiposity in women, while stable high pattern was associated with higher odds of overweight (OR = 1.88; 95% CI: 1.11, 3.19) in men. Additionally, high compared to the lowest household wealth was associated with high odds of obesity and central adiposity in both sexes.

**Conclusion:**

Stable high and upwardly mobile education patterns are associated with higher odds of obesity and central adiposity in women while the stable high pattern was associated with higher odds of overweight in men.

## Introduction

Overweight and obesity have reached epidemic proportions in most regions of the world, including sub-Saharan Africa [1, 2]. With increasing prevalence of overweight and obesity in low and middle-income countries (LMICs), the World Health Organization (WHO) has indicated that a double burden of communicable and non-communicable diseases in the near future in LMICs is imminent [3]. Since increasing prevalence of overweight and obesity could differ based on several factors including sex, biosocial, sociocultural, economics, biological, and environmental factors, it is crucial to understand the factors that contribute to their occurrence in LMICs in order to tailor efficient and cost-effective preventive programmes and policies in their prevention [4, 5].

Socioeconomic status (SES), is a consistent predictor of population morbidity and mortality [6, 7]. Some SES markers include education, intergenerational education mobility and income/wealth [6, 8, 9]. Education has been cited as one of the most important markers of SES that affect individuals’ health [6–9]. However, the effect of intergenerational educational mobility on individuals’ health is scarcely examined in many LMICs settings. Intergenerational educational mobility is largely defined as the change in the level of education between parent(s) and their children and describes individuals’ experiences in relation to an achieved position compared to their parents [8, 10]. Previous studies have shown that different childhood or initial socioeconomic circumstances might influence current and future health outcomes and inequalities [7, 8, 10, 11]. Studies conducted mostly in high-income countries that used education to define lifetime SES found individuals with high levels of education compared with their parents had better health outcomes than those who had lower education compared with their parents [8, 10, 11]. Wealth has been found to be inversely associated with obesity in developed countries [5, 12], but has shown to be positively associated with obesity in developing countries [2, 5, 13].

Few studies have explored the mechanism by which intergenerational education mobility influences health risks in low and middle-income countries, and Ghana is no exception [14, 15]. However, these studies have used BMI as a continuous variable, which limits the ability to differentiate between categories of BMI. Hence, the associations for overweight or obesity have not been distinctively made. Furthermore, categorizing BMI into obese and non-obese where underweight and overweight are grouped as non-obese could be problematic as the two categories may produce different health effects [16, 17]. Waist circumference is found to be an important marker of central adiposity and could be used independently to predict cardiometabolic diseases in different populations [18, 19]. Thus, previous studies usage of only BMI to determine obesity may limit the appropriate capturing of central adiposity. Lastly, previous studies have used sub-populations that often do not represent the population of interest and limits the generalizability of the findings. To fill the gap in these previous studies, this study uses Wave 2 data of the World Health Organization’s Study on global AGEing and adult health (WHO-SAGE) to examine the association between markers of current and lifetime SES and different BMI categories in a representative sample of adult men and women in Ghana. We also conduct sub-analyses using current sub-Saharan Africa (SSA) population waist circumference optimal cut-offs for which individuals are at increased risk of cardiometabolic diseases [18].

## Methods

### Study population

Data from SAGE Ghana Wave 2 (2014–2015) was used. This is a longitudinal dataset on the health and well-being of adult populations aged ≥50 years for six countries: China, Ghana, India, Mexico, Russian Federation, and South Africa [20]. For comparison, the study also collects sample data from younger adults aged 18–49 years. In Ghana, SAGE collected individual-level data from a nationally representative sample of households including older adults using a stratified, multistage cluster design. The primary sampling units were stratified by region and location of residence of a household (urban/rural) and the samples were selected from 250 enumeration areas. WHO SAGE was approved by the WHO Ethics Review Committee (reference number RPC149) with local approval from the University of Ghana Medical School Ethics and Protocol Review Committee (Ghana). Further information on WHO SAGE can be found at http://www.who.int/healthinfo/sage/cohorts/en/.

The individual questionnaire responses used in the study covered the following domains: socio-demographic characteristics, work history and benefits; health state descriptions; anthropometrics, performance tests and biomarkers; risk factors and preventive health behaviour; chronic conditions and health services coverage; health care utilisation; social cohesion; subjective well-being and quality of life; impact of caregiver; and the interviewer’s assessment. Of the 4,735 survey respondents, 229 had missing data for height, 227 for weight and 228 for waist circumference. Also, biologically implausible values (BIV) (height <100cm or >200 cm and weight <30.0 kg or >250.0 kg and waist circumference < 25.0 cm or > 220 cm) were excluded using the listwise deletion [21, 22]. In total, 246 (5.2%) and 25 (0.5%) observations were excluded due to missing anthropometric measurements and BIVs of height. Consequently, data from 4,464 participants who had complete responses formed the analytical sample for the study.

### Outcome variables

In the WHO SAGE data, anthropometric measurements of body weight, height, and waist circumference of respondents were taken by trained assessors using standard protocols [23, 24]. Pregnant women were exempted from weight and waist circumference measurements [23]. Respondents’ height was converted from centimeters to meters, and BMI was calculated as a person’s weight in kilograms divided by the square of their height in meters (kg/m^2^). BMI was classified into four categories and weighted prevalence estimated: underweight, BMI <18.50kg/m^2^; normal/healthy BMI, BMI ≥18.50–24.99 kg/m^2^; overweight, 25.00–29.99kg/m^2^; and obesity as BMI ≥30.00 kg/m^2^ [25, 26]. We determined central adiposity using the current waist circumference optimal cut-offs for which individuals would be at increased risk for cardiometabolic diseases within the sub-Saharan Africa (SSA) population [18]. Thus, waist circumference (WC) cut-off of WC ≥ 81.2cm for men, WC ≥80.0cm for women and population WC ≥81.1cm were categorized as central adiposity otherwise, normal.

### Explanatory variables

#### SES variables

The selection of SES measures follows previous literature [6, 8, 10, 27]. These were parental and individual education, intergenerational educational mobility, and household wealth status. SAGE collected information on the respondent (individual) and parental highest level of education. The structure of the Ghanaian Educational System before and after independence in 1957 has been subjected to several structural and funding changes with significant debates around the number of years students should spend in the Senior Secondary/High School (SSS/SHS) [28]. In addition to reducing the number of years spent in pre-tertiary education, improving the quality of education and creating universal access to education, another principal aim of the SSS/SHS educational structural reforms was to enhance economic growth by ensuring that SSS/SHS school leavers would develop skills to secure jobs in the labour market when they exited the school system before tertiary education [28, 29]. Thus, the SSS/SHS would act as a better and preferable education level for entry into the labour market. Many structural reforms have occurred between 1961 and 1995. In 1995, the Free Compulsory Universal Basic Education (FCUBE) was introduced to improve the quality of education with basic education consisting of nine years. Universal Basic Education is made up of six (6) years of primary school education and three (3) years of Junior Secondary School (JSS) [28]. Between 2002 and 2008, this was changed to 11 years of free basic education to include two years of kindergarten education, and then four years of senior secondary school education. Since 2017, Ghana has embarked on free education from kindergarten to SHS [30]. While male participation in education has consistently been high, that of females has gradually increased, although with some major barriers [31].

On the basis that SSS/SHS would act as the minimum level of education required for entry into the labour market [29], and also due to fewer data observations for college or tertiary education, we divided the highest education completed into two groups. Thus, both parental and individual educational levels were grouped into low education where the highest level of education was less than a secondary or high school, and high education where a person completed secondary/ high school and above.

**Fig 1:**
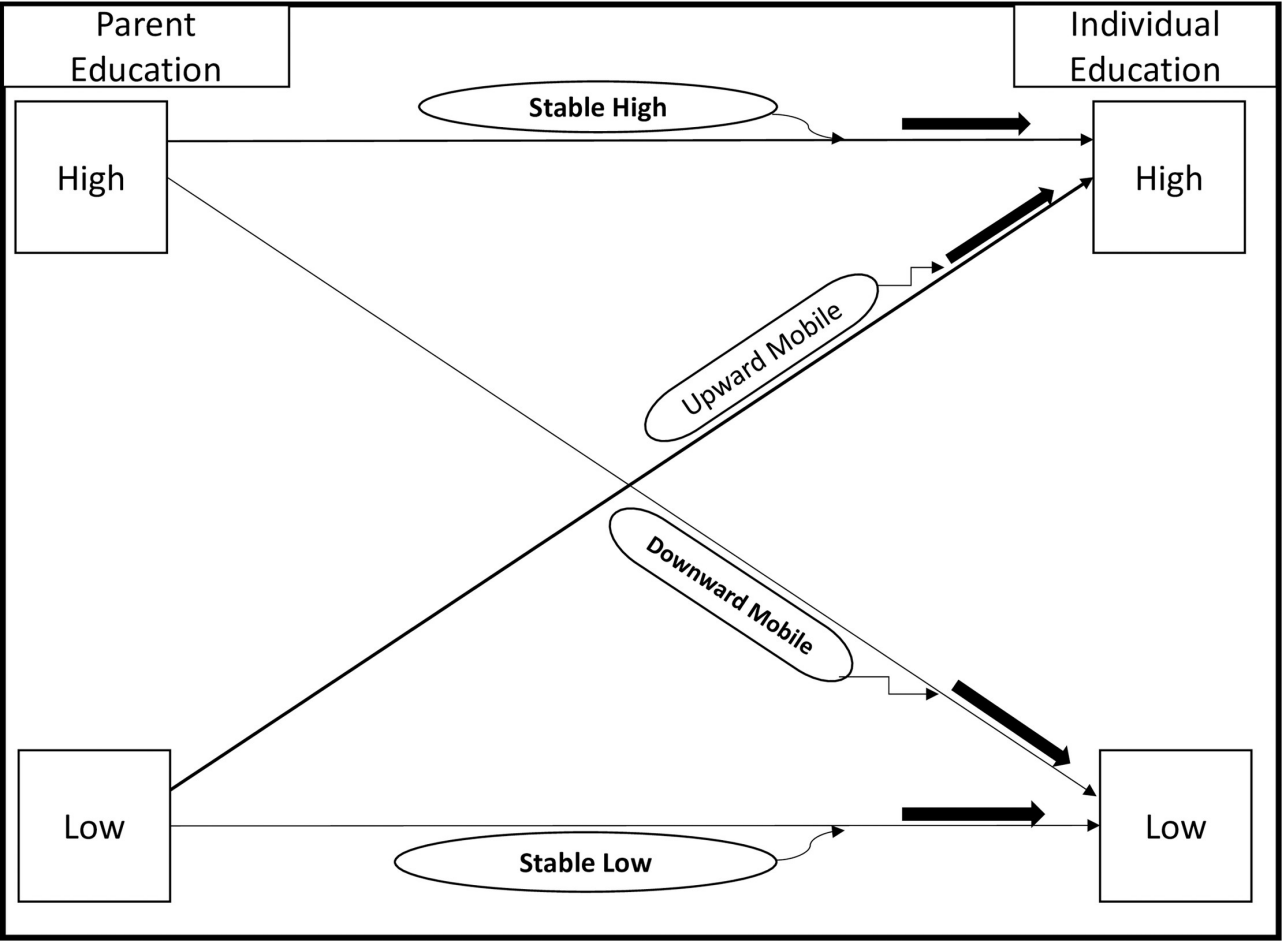
Schematic representation of intergenerational education mobility tracking possibilities from parental education to respondents' education in the WHO-SAGE study.

We used education to define the lifetime SES variable: intergenerational educational mobility was selected as studies have shown that education is reliable and vital in determining long-term social class when studying health risk factors [6–8]. Intergenerational educational mobility was assigned and coded as follows: 1) stable low if both parents and individual education were low; 2) stable high if both parents and individual education were high; 3) upwardly mobile if parents had low education and individual education was high; and, 4) downwardly mobile if parents had high education and individual education was low (Fig 1).

Household wealth index was constructed using principal component analysis from a total of 22 assets/ characteristics/ items converting these into wealth quintiles [32, 33]. The items included household ownership of durable assets (example; radio, television, and refrigerator), dwelling characteristics (example; type of floor and wall material) and access to utilities (example; electricity, improved water and having improved sanitation facility). Quintile one was the lowest quintile; two represented low; three for moderate; four was high, and five was the highest quintile representing highest household wealth status. This served as a proxy for household economic status [32–34].

#### Covariates

Covariates included age, place of residence, marital status, alcohol intake, smoking status, daily fruit and vegetable intake, and weekly physical activity levels. In this study, age was specified in 10-year intervals except for ages 18–29 due to fewer observations based on the stated sampling strategy where the younger adult population was selected for comparison purposes, not to be nationally representative of these younger age groups. Rural/urban residence was defined by localities, where population size less than 5,000 was classified as rural and any larger localities classified as urban. Marital status was coded as (1) for single, (2) for married/ cohabiting and (3) for divorced/ separated/ widow/ widower. Respondents’ smoking status was coded as (1) for “current smoker”, (2) “former smoker” and (3) for “never smoked before”; and alcohol consumption status was also coded as (1) for “current alcohol drinker”, (2) “former alcohol drinker” and (3) for “never drunk alcohol before”.

Daily fruit and vegetable servings were categorized according to the NCD Global Monitoring Framework specifications and recommended by the joint WHO/FAO Panel on Diet, Nutrition and Chronic Disease Prevention [35]. Following these recommendations, a serving of fruit or vegetables was equivalent to 80 grams. Respondents were determined to have met the standard recommendation if they ate five or more servings of fruits and vegetables per day (equivalent to 400 grams). The level of physical activity was determined by the total metabolic equivalents of task (MET) minutes per week using the Global Physical Activity Questionnaire (GPAQ) built into the SAGE interview instrument [36]. Meeting the recommended total physical activity was defined as engaging in activities including work, during transport and leisure time for at least 150 minutes of moderate-intensity activity per week or 75 minutes of vigorous-intensity activity per week. The Cronbach’s alpha for the 15 items covering work, travel to and from places, and recreational activities that measured physical activity was 85.4% in this sample.

### Statistical analysis

The prevalence estimates for each BMI category and central adiposity were computed as percentages using the total sample size separately for men, women and the total population as the denominator. In the estimation process, we used post-stratified person weights to account for the differential probabilities of selection, nonresponse, and non-coverage/under-coverage. Using these weights, we were able to estimate prevalence that reflects the true distribution of the BMI categories and central adiposity in the population aged 50 years and older [37]. Due to the categorical nature of the outcome variables in this study, we used Pearson chi-squared test or Fisher’s exact test where appropriate, to examine the associations between all BMI categories, the two categories of waist circumference and the mediators.

Taking the post-stratified person weights into account, we fitted multilevel multinomial and binomial logistic models using the Generalized Latent Linear and Mixed Model (GLLAMM) to determine the association between all the SES factors with the four-level outcome variable of BMI category (underweight, normal weight, overweight, and obese) [38]. Normal/healthy weight category was used as the base/reference group. We estimated the exponentiated form of the coefficients and therefore presented the results in terms of multinomial and binomial log-odds (logit) instead of relative risk ratios [39]. Thus, we estimated the odds of being underweight, overweight, obese or having “at risk” WC compared to having normal BMI.

Ignoring the post-stratified sampling weights, inability to account for both unobserved variables and dependence among observations are major identification problems, which could lead to biased estimations in this study. GLLAMM accounting for the sampling weights of the observation and the latent trait in GLLAMM helps to account for unobservable variables leading to unbiased estimates. The SAGE has a hierarchical structure, in which participants were nested within survey clusters. To adjust for dependence because of the clustering in the multivariable analyses, we used GLLAMM and included individual random effects in the model to estimate the magnitude and determine the significance of clustering using Intra Class Correlation (ICC). We determined the ICC as the ratio of the variance at the cluster level to the sum of the variance at the individual and the cluster levels [16]. All analyses were performed using STATA v.15 (Stata Corp., College Station, Texas, USA).

Two models for each sex and each SES factor were developed and were adjusted for covariates that were identified as confounders. Confounders were determined to be covariates if they were associated with the outcome and when included in the model resulted in a change in the parameter estimate by 10% or more [40]. Confounders were age, ethnicity and marital status (included in model 1 for each sex), while location, physical activity, alcohol, and smoking status, as well as fruit and vegetable intake, were used as potential mediators [27]. Both confounders and potential mediators were included in the final model (model 2) for each sex. We reported the results of a third model (Model 3 in Supplementary Table 1) in which household wealth status was included in model 2 separately for parental and individual education, and intergenerational education mobility, to test which of these SES factors remained associated with the categories of BMI. We assessed multicollinearity using variance inflation factors. There was no variance inflation factor higher than 10, suggesting no multicollinearity in both models 1 and 2 [41].

**Table 1:** Characteristics of the sample, by sex in WHO SAGE Ghana Wave 2 (2014/15).

		Men		Women		Population	
		(n)	%	(n)	%	(n)	%
Total Population		1854	41.5	2610	58.5	4464	100
Age [years (SD)]		58.1 (16.8)		56.4 (16.2)		57.1 (16.5)	
Mean Weight [kg (SD)]		64.5 (11.5)		66.0 (16.0)		65.3 (14.2)	
Mean Height [m (SD)]		1.68 (7.59)		1.59 (7.49)		1.63 (8.63)	
Mean Waist Circumference [cm (SD)]		79.7 (12.6)		86.6 (17.7)		83.4 (15.9)	
Mean BMI [kg/m^2^ (SD)]		22.9 (3.7)		25.9 (6.1)		24.5 (5.4)	
BMI Categories	Normal Weight	1220	65.8	1248	47.8	2468	55.3
	Underweight	250	13.5	248	9.5	498	11.2
	Overweight	320	17.3	640	24.5	960	21.5
	Obese	64	3.5	474	18.2	538	12.1
^‡^Central Adiposity (WC ≥ optimal cut-point)		864	46.6	1898	72.7	2762	61.9
Age (years)	18–29	160	8.6	207	7.9	367	8.2
	30–39	107	5.8	216	8.3	323	7.2
	40–49	189	10.2	235	9.0	424	9.5
	50–59	443	23.9	805	30.8	1248	28.0
	60–69	485	26.2	577	22.1	1062	23.8
	≥ 70	470	25.4	570	21.8	1040	23.3
Parental Education	Low	1619	87.3	2205	84.5	3824	85.7
	High	235	12.7	405	15.5	640	14.3
Individual Education	Low	1111	59.9	2047	78.4	3158	70.7
	High	743	40.1	563	21.6	1306	29.3
Intergenerational Education Mobility	Stable Low	1071	57.8	1906	73.0	2,977	66.7
	Stable High	195	10.5	264	10.1	459	10.3
	Upward Mobility	548	29.6	299	11.5	847	19.0
	Downward Mobility	40	2.2	141	5.4	181	4.1
Household Wealth Quintile	Lowest	329	17.8	263	10.1	592	13.3
	Low	421	22.7	538	20.6	959	21.5
	Moderate	342	18.5	609	23.3	951	21.3
	High	390	21.0	615	23.6	1005	22.5
	Highest	372	20.1	585	22.4	957	21.4

^**‡**^ sub-Saharan African waist circumference (WC) cut-off point for men (WC≥81.2cm); women (WC≥80.0cm); population level (WC≥81.1cm) [18]

## Results

Table 1 presents the characteristics of the study population. In this sample, there were more women (58.5%) compared to men (41.5%). The mean age (standard deviation, SD) of the population was of 57.1 years (SD: 16.5) with a mean height of 1.63m (SD: 8.63), weight of 65.3kg (SD: 14.2), waist circumference of 83.4cm (15.9) and BMI of 24.5kg/m^2^ (SD: 5.4). While about 40% of men had high education, only about 22% of women had the same level of education.

The weighted prevalence across the four BMI categories and central adiposity are shown in Table 2. Accounting for sampling weights, 7.2% (95% CI: 6.0%, 8.7%) were underweight; 55.2% (95% CI: 52.1%, 58.2%) were normal weight; 24.7% (95% CI: 22.3%, 27.2%) were overweight; 12.9% (95% CI: 11.2%, 14.8%) were obese by BMI measurement, and 61.9% (95% CI: 60.4%, 63.3%) had elevated central adiposity by waist circumference measurement. Thus, the prevalence of central adiposity that increases cardiometabolic risk was higher compared to the combined prevalence of overweight and obesity in both men and women (Table 2).

**Table 2:** Prevalence (%) of Body Mass Index (BMI) categories and central adiposity (Waist Circumference ≥ cut-off point) with 95% Confidence Interval in Ghana’s adult population (2014/15).

		Underweight	Normal weight	Overweight	Obese	^‡ ‡^ Central Adiposity (WC ≥ optimal cut-point)
**Men**	^**‡**^**Weighted**	9.1 (6.9, 11.9)	66.2 (61.4, 70.7)	20.7 (17.1, 24.9)	3.9 (2.5, 6.2)	42.7 (38.7, 46.7)
	**Unweighted**	13.4 (12.0, 15.1)	65.8 (63.6, 67.9)	17.3 (15.6, 19.1)	3.5 (2.7, 4.4)	46.6 (44.3, 48.9)
^**‡**^**Parental Education**	Low	9.5 (7.1, 12.5)	66.0 (61.1, 70.5)	20.7 (16.7, 25.3)	3.8 (2.4, 5.9)	42.6 (38.2, 47.2)
	High	8.0 (84.4, 14.0)	66.8 (56.2, 75.9)	20.8 (13.0, 29.8)	4.4 (2.0, 9.5)	42.8 (34.1, 51.8)
^**‡**^**Individual Education**	Low	5.6 (3.5, 8.9)	68.4 (61.4, 74.7)	20.6 (16.2, 25.7)	5.4 (2.9, 9.7)	42.6 (37.0, 48.4)
	High	5.6 (3.5, 8.9)	68.4 (61.4, 74.7)	20.6 (16.2, 25.7)	5.4 (2.9, 9.7)	42.6 (37.0, 48.4)
^**‡**^**Intergenerational Education Mobility**	Stable Low	12.1 (8.7, 16.6)	64.1 (58.1, .69.7)	21.1 (16.0, 27.2)	2.6 (1.3, 5.2)	43.7 (37.9, 49.7)
	Stable High	5.9 (2.9, 10.8)	67.7 (55.2, 78.1)	21.2 (13.6, 31.6)	5.1 (2.2, 11.6)	45.0 (35.6, 54.7)
	Upwardly	5.4 (3.3, 8.7)	68.9 (61.6, 75.5)	20.1 (15.0, 26.4)	5.6 (2.9, 10.4)	40.8 (34.1, 48.0)
	Downwardly	14.1 (8.0, 23.6)	64.1 (44.6, 79.8)	19.6 (12.0, 26.6)	2.1 (1.0, 6.9)	35.9 (18.8, 50.7)
^**‡**^**Household Wealth Quintiles**	Lowest	11.5 (7.0, 18.5)	74.0 (66.7, 80.2)	13.7 (8.5, 21.2)	0.8 (0.2, 3.5)	29.2 (21.1, 38.9)
	Low	8.6 (5.0, 14.3)	76.0 (68.5, 82.1)	12.6 (8.2, 19.0)	2.8 (1.0, 6.2)	41.0 (32.2, 50.5)
	Moderate	13.6 (8.7, 20.5)	62.0 (52.7, 70.4)	22.2 (14.7, 32.3)	2.2 (0.9, 5.6)	50.7 (40.7, 60.6)
	High	10.2 (6.2, 16.4)	65.2 (56.8, 72.7)	20.5 (14.3, 26.4)	4.1 (1.9, 8.6)	39.8 (32.1, 48.1)
	Highest	4.2 (1.5, 10.3)	60.2 (48.9, 70.6)	28.4 (21.4, 36.6)	7.1 (3.4, 14.2)	47.2 (38.5, 56.0)
** Women**	^**‡**^**Weighted**	9.0 (6.6, 12.1)	45.9 (42.6, 49.2)	28.0 (25.2, 31.0)	20.4 (17.8, 23.4)	70.9 (67.1, 74.4)
	**Unweighted**	9.5 (8.4, 10.7)	47.8 (45.9, 49.7)	24.5 (22.9, 26.2)	18.2 (16.7, 19.7)	72.7 (71.0, 74.4)
^**‡**^**Parental Education**	Low	5.3 (4.0, 7.0)	47.9 (44.2, 51.5)	26.9 (23.8, 30.3)	19.9 (17.1, 23.0)	71.7 (67.5, 75.6)
	High	6.8 (3.8, 10.9)	40.2 (33.5, 47.2)	31.0 (25.0, 37.7)	22.0 (16.5, 28.7)	68.3 (60.7, 75.0)
^**‡**^**Individual Education**	Low	5.7 (4.2, 7.5)	47.3 (43.4, 51.3)	27.8 (24.6, 31.3)	19.2 (16.1, 22.7)	71.6 (67.4, 75.5)
	High	5.7 (3.2, 9.9)	42.8 (37.0, 48.7)	28.4 (22.7, 34.8)	23.2 (18.3, 28.8)	69.2 (62.2, 75.4)
^**‡**^**Intergenerational Education Mobility**	Stable Low	6.0 (4.4, 8.0)	48.3 (44.2, 52.4)	26.3 (23.0, 29.9)	19.4 (16.2, 23.2)	70.9 (66.3, 75.1)
	Stable High	8.5 (4.3, 16.1)	40.2 (32.4, 48.5)	27.2 (20.1, 35.5)	24.2 (17.0, 33.3)	64.1 (54.5, 72.7)
	Upwardly	2.3 (1.0, 5.5)	45.9 (36.8, 55.2)	29.8 (20.9, 40.5)	22.0 (15.7, 29.9)	75.2 (65.4, 83.0)
	Downwardly	3.5 (1.1, 9.2)	40.2 (28.7,50.8)	38.8 (28.1, 48.7)	17.6 (11.2, 26.4)	76.9 (64.6, 85.8)
^**‡**^**Household Wealth Quintiles**	Lowest	6.9 (3.6, 13.1)	56.9 (45.5, 67.6)	28.8 (19.9, 39.7)	7.4 (2.8, 14.2)	55.9 (44.1, 67.1))
	Low	7.4 (4.3, 12.6)	53.0 (44.3, 61.5)	26.4 (19.2, 35.3)	13.1 (7.3, 20.2)	68.1 (60.2, 75.1)
	Moderate	5.4 (3.6, 8.2)	52.3 (45.2, 59.4)	27.4 (21.7, 33.8)	14.9 (10.5, 20.7)	73.4 (66.7, 79.3)
	High	7.9 (4.6, 13.3)	37.1 (31.1, 43.5)	32.2 (26.1, 38.9)	22.8 (17.9, 28.6)	73.4 (66.2, 79.5)
	Highest	2.2 (1.0, 5.1)	40.8 (34.0, 48.1)	25.4 (20.1, 31.4)	31.6 (25.2, 38.7)	73.6 (65.7, 80.3)
**Total Population**	^**‡**^**Weighted**	7.2 (6.0, 8.7)	55.2 (52.1, 58.2)	24.7 (22.3, 27.2)	12.9 (11.2, 14.8)	61.9 (60.4, 63.3)
	**Unweighted**	11.2 (10.3, 12.1)	55.3 (53.8, 56.7)	21.5 (20.3, 22.7)	12.1 (11.1, 13.0)	58.0 (54.8, 61.1)

^‡^Weighted Prevalence:—Post-stratified person weight applied. Only weighted prevalence is presented for parents and individual education, intergenerational education mobility and household wealth quintiles

^‡ ‡^ sub-Sahara waist circumference optimal cut-off point for men (WC≥81.2cm); women (WC≥80.0cm); population level (WC≥81.1cm) [18]

Also, the prevalence of obesity exceeded that of underweight in the population. The prevalence of overweight, obesity and central adiposity was higher in women, particularly in all the high SES categories. Tables 3 (men) and 4 (women) show that all the SES markers were associated with BMI and central adiposity. In addition, SES status was found to be associated with covariates such as location and in some cases alcohol intake, low fruit and vegetable intake, and physical activity levels. These covariates were used as mediators in the regression analyses.

**Table 3:** Prevalence of covariates (mediators) by education categories and household wealth in men.

	BMI			Central Adiposity	Urban Location	Smoking Status		Alcohol Status		Below recommended ^‡^ FV intake	Below recommended physical activity levels
	Underweight	Overweight	Obese	(WC≥81.2cm)		Current smoker	Former smoker	Current drinker	Former drinker		
**Parental Education**											
Low	231	265	52	752	506	155	73	589	128	864	887
High	19	55	12	112	137	15	18	89	22	120	118
P*-value*	<0.01			<0.01	<0.01	0.04		0.61		0.53	0.21
**Individual Education**			** **	** **							
Low	171	166	30	499	302	124	44	383	82	583	588
High	79	154	34	365	341	46	47	295	68	401	417
P*-value*	<0.01			0.08	<0.01	<0.01		0.01		0.54	0.18
**Intergenerational Education Mobility**											
Stable Low	165	160	29	485	284	121	41	370	78	562	569
Stable High	13	49	11	98	119	12	15	76	18	99	99
Upwardly	66	105	23	267	222	34	32	219	50	302	318
Downwardly	6	6	1	14	18	3	3	13	4	21	19
P*-value*	<0.01			0.18	<0.01	<0.01		0.15		0.68	0.14
**Household Wealth Quintiles**		** **	** **	** **							
Poorest	59	38	4	100	21	61	14	127	21	161	160
Poor	63	50	7	194	55	37	17	148	20	237	221
Moderate	60	49	5	168	108	29	18	126	43	191	199
Rich	49	73	21	181	203	28	23	153	29	218	229
Richest	19	110	27	221	256	15	19	124	37	177	196
P*-value*	<0.01			<0.01	<0.01	<0.01	<0.01	<0.01	<0.01	0.03	0.04

^‡^ FV: Fruits and vegetable intake

**Table 4:** Prevalence of covariates (mediators) by education categories and household wealth in women.

	BMI			Central Adiposity	Urban Location	Smoking Status		Alcohol Status		Below recommended ^‡^ FV intake	Below recommended physical activity levels
	Underweight	Overweight	Obese	(WC≥80.0cm)		Current smoker	Former smoker	Current drinker	Former drinker		
**Parental Education**											
Low	232	517	352	1588	902	17	22	273	136	1158	1319
High	16	123	122	310	276	4	1	54	33	204	263
P*-value*	<0.01			0.03	<0.01	0.30		0.26		0.45	0.05
**Individual Education**			** **	** **							
Low	221	485	311	1462	815	19	22	263	130	1091	1236
High	27	155	163	436	363	2	3	64	39	271	346
P*-value*	<0.01			<0.01	<0.01	0.05		0.59		0.03	0.66
**Intergenerational Education Mobility**											
Stable Low	216	438	275	1353	733	16	22	237	114	1028	1136
Stable High	11	76	86	201	194	1	1	28	17	141	163
Upwardly	16	79	77	235	169	1	0	36	22	130	183
Downwardly	5	47	36	109	82	3	0	26	16	63	100
P*-value*	<0.01			0.01	<0.01	0.09		0.04		<0.01	0.06
**Household Wealth Quintiles**		** **	** **	** **							
Poorest	31	50	14	152	22	0	6	28	9	132	155
Poor	71	106	39	363	112	4	5	76	28	311	338
Moderate	76	139	71	449	244	7	6	62	52	327	361
Rich	50	175	147	465	363	6	1	77	39	312	370
Richest	20	170	203	469	437	4	5	84	41	280	358
P*-value*	<0.01			<0.01	<0.01	0.11		0.03		0.01	0.74

^‡^ FV: Fruits and vegetable intake

Results of the multivariable analyses for the SES markers associated with the BMI categories and central adiposity are shown in Tables 5 and 6 for men and women, respectively. Model 1 in men showed that high parental education was associated with lower odds of underweight but higher odds of both overweight and obesity. In the fully adjusted model, the association was no longer significant except for those in the underweight (OR = 0.40; 95% CI: 0.16, 0.97) and overweight (OR = 1.66; 95% CI: 1.05, 2.65) categories. Parental education was not associated with central adiposity in men. In women, parental education was significantly associated with BMI and central adiposity. High parental education was significantly associated lower odds of underweight (OR = 0.31; 95% CI: 0.12, 0.75), but higher odds of overweight (OR = 1.84; 95% CI: 1.29, 2.62), obesity (OR = 2.59; 95% CI: 1.72, 3.91) and central adiposity (OR = 1.51; 95% CI: 1.04, 2.19) in the fully adjusted model.

**Table 5:** Odds ratios (95% confidence intervals, CI) of being underweight, overweight, or obese according to markers of socioeconomic status in men in Ghana (2014/15).

Men	^1^Model 1			^2^Model 2			^1^Model 1	^2^Model 2
	BMI (Ref: normal/healthy BMI)						Central Adiposity (^3^WC≥81.2cm)	
	Underweight OR [95% CI]	Overweight OR [95% CI]	Obese OR [95% CI]	Underweight OR [95% CI]	Overweight OR [95% CI]	Obese OR [95% CI]	Central Adiposity OR [95% CI]	Central Adiposity OR [95% CI]
**Parental Education**								
Low	1.00	1.00	1.00	1.00	1.00	1.00	1.00	1.00
High	0.35 (0.14, 0.87) *	2.06 (1.31, 3.25) **	2.80 (1.16, 5.74) *	0.40 (0.16, 0.97) *	1.66 (1.05, 2.65) *	1.96 (0.82, 4.67)	1.30 (0.86, 1.97)	1.09 (0.72, 1.67)
**Individual Education**								
Low	1.00	1.00	1.00	1.00	1.00	1.00	1.00	1.00
High	0.70 (0.47, 1.03)	1.71 (1.21, 2.43) **	1.64 (0.86, 3.16)	0.79 (0.53, 1.17)	1.41 (0.99, 2.10)	1.28 (0.65, 2.52)	1.25 (0.94, 1.64)	1.09 (0.82, 1.45)
**Intergenerational Education Mobility**								
Stable Low	1.00	1.00	1.00	1.00	1.00	1.00	1.00	1.00
Stable High	0.28 (0.11, 0.77) **	2.52 (1.50, 4.23) ***	3.30 (1.27, 7.55) **	0.32 (0.12, 0.90) *	1.88 (1.11, 3.19) *	2.06 (0.80, 54.29)	1.40 (0.88, 2.23)	1.11 (0.69, 1.79)
Upwardly	0.82 (0.54, 1.23)	1.60 (1.09, 2.37) **	1.24 (0.61, 2.50)	0.91 (0.61, 1.37)	1.36 (0.92, 2.02)	1.04 (0.49, 2.22)	1.23 (0.91, 1.65)	1.11 (0.85, 1.50)
Downwardly	1.03 (0.19, 3.63)	3.70 (0.76, 7.00)	0.35 (0.04, 3.38)	1.16 (0.19, 3.10)	3.34 (0.85, 6.21)	0.35 (0.03, 1.88)	1.75 (0.57, 3.39)	1.70 (0.57, 4.06)
**Household Wealth Quintile**								
Lowest	1.00	1.00	1.00	1.00	1.00	1.00	1.00	1.00
Low	0.60 (0.38, 0.96) *	1.14 (0.56, 2.31)	1.00 (0.20, 3.12)	0.60 (0.48, 1.23)	0.93 (0.53, 1.67)	0.69 (0.13, 2.77)	2.21 (1.46, 3.33) ***	1.89 (1.15, 3.11) **
Moderate	0.57 (0.34, 0.94) *	1.32 (0.62, 2.81)	2.38 (0.51, 5.62)	0.67 (0.41, 1.04)	1.45 (0.84, 2.52)	2.03 (0.43, 5.07)	2.37 (1.59, 3.55) ***	1.92 (1.23, 3.00) **
High	0.49 (0.30, 0.82) **	2.20 (1.13, 4.30) *	4.28 (2.10, 9.78) ***	0.65 (0.38, 1.11)	1.52 (0.83, 2.82)	3.74 (1.53, 8.66) *	2.49 (1.58, 3.92) ***	2.06 (1.34, 3.15) ***
Highest	0.20 (0.11, 0.38) ***	3.06 (1.64, 6.85) ***	5.36 (3.30, 10.99) ***	0.24 (0.11, 0.49) ***	1.83 (1.01, 3,34) *	4.52 (2.51, 9.95) **	4.46 (2.87, 6.92) ***	2.98 (1.83, 4.83) ***

*p<0.05

**p<0.01

***p<0.001

^1^Model 1- Adjusted for confounders including age, marital status and ethnicity

^2^Model 2- Adjusted for confounders and potential mediators including rural/urban location, whether a respondent was current smoker, former smoker or never smoked, regularly/ currently drinks

alcohol, former or has never drunk alcohol, met the recommended daily fruit and vegetable intake level, and met the recommended weekly level of physical activity.

^3^sub-Sahara waist circumference optimal cut-off point for men (WC≥81.2cm) [18]

**Table 6:** Odds ratios (95% confidence intervals, CI) of being underweight, overweight, or obese according to markers of socioeconomic status in women in Ghana (2014/15).

Women	^1^Model 1			^2^Model 2			^1^Model 1	^2^Model 2
	BMI (Ref: normal/healthy BMI)						Central Adiposity (^3^WC≥80.0cm)	
	Underweight OR [95% CI]	Overweight OR [95% CI]	Obese OR [95% CI]	Underweight OR [95% CI]	Overweight OR [95% CI]	Obese OR [95% CI]	Central Adiposity OR [95% CI]	Central Adiposity OR [95% CI]
**Parental Education**								
Low	1.00	1.00	1.00	1.00	1.00	1.00	1.00	1.00
High	0.28 (0.11, 0.71) **	2.04 (1.46, 2.84) ***	3.34 (2.19, 5.10) ***	0.31 (0.12, 0.75) **	1.84 (1.29, 2.62) ***	2.59 (1.72, 3.91) ***	1.77 (1.23, 2.54) **	1.51 (1.04, 2.19) *
**Individual Education**								
Low	1.00	1.00	1.00	1.00	1.00	1.00	1.00	1.00
High	0.50 (0.28, 0.89) *	1.52 (1.13, 2.05) **	2.71 (1.90, 3.87) ***	0.56 (0.32, 1.00)	1.34 (0.99, 1.83)	2.05 (1.45, 2.91)	1.87 (1.35, 2.59) ***	1.62 (1.16, 2.27) **
**Intergenerational Education Mobility**								
Stable Low	1.00	1.00	1.00	1.00	1.00	1.00	1.00	1.00
Stable High	0.18 (0.06, 0.52) **	2.16 (1.42, 3.31) ***	4.54 (2.77, 7.43) ***	0.21 (0.07, 0.61) **	1.87 (1.19, 2.93) **	3.15 (1.96, 5.05) ***	2.13 (1.31, 3.49) **	1.75 (1.03, 2.98) *
Upwardly	0.67 (0.17, 1.59)	1.32 (0.88, 1.98)	2.18 (1.44, 3.28) ***	0.73 (0.38, 1.38)	1.17 (0.78, 1.77)	1.71 (1.13, 2.60) **	1.80 (1.20, 2.69) **	1.60 (1.08, 2.37) *
Downwardly	0.43 (0.11, 1.63)	2.06 (1.22, 3.46) **	2.86 (1.61, 5.11) ***	0.42 (0.12, 1.45)	1.92 (1.11, 3.32) **	2.42 (1.30, 4.53) **	1.60 (0.91, 2.81)	1.43 (0.82, 2.50)
**Household Wealth Quintile**								
Lowest	1.00	1.00	1.00	1.00	1.00	1.00	1.00	1.00
Low	1.09 (0.64, 1.86)	1.26 (0.80, 1.99)	1.39 (0.56, 3.45)	1.01 (0.60, 1.72)	1.19 (0.75, 1.88)	1.19 (0.47, 2.98)	1.51 (1.01, 2.27) *	1.38 (0.92, 2.08)
Moderate	0.99 (0.59, 1.67)	1.61 (1.03, 2.51) *	2.94 (1.84, 7.03) *	1.21 (0.69, 2.11)	1.44 (0.91, 2.28)	2.02 (0.84, 4.89)	2.10 (1.35, 3.27) ***	1.79 (1.15, 2.79) **
High	0.96 (0.54, 1.70)	2.74 (1.74, 4.29) ***	4.91 (2.27, 10.15) ***	1.10 (0.60, 2.02)	2.30 (1.42, 3.71) ***	3.75 (1.93, 7.68) ***	2.36 (1.51, 3.69) ***	1.86 (1.18, 2.93) **
Highest	0.62 (0.31, 1.24)	4.31 (2.64, 7.06) ***	6.29 (2.85, 16.77) ***	0.76 (0.37, 1.58)	3.44 (2.02, 5.88) ***	4.48 (3.49, 9.59) ***	3.19 (1.96, 5.20) ***	2.34 (1.42, 3.85) ***

*p<0.05

**p<0.01

***p<0.001

^1^Model 1- Adjusted for confounders including age, marital status and ethnicity

^2^Model 2- Adjusted for confounders and potential mediators including rural/urban location, whether a respondent was current smoker, former smoker or never smoked, regularly/currently drinks

alcohol, former or has never drunk alcohol, met the recommended daily fruit and vegetable intake level, and met the recommended weekly level of physical activity.

^3^sub-Sahara waist circumference cut-off point for women (WC≥80.0cm) [18]

While no significant association was found between individual education and BMI or central adiposity in men, a significant association was found in women who were obese and women with central adiposity. High compared to low individual education was associated with higher odds of obesity (OR = 2.05; 95% CI: 1.45, 2.91) and central adiposity (OR = 1.62; 95% CI: 1.16, 2.27). When individual education was independently adjusted for parental education in the full model, high individual education was found to be associated with higher odds of obesity (OR = 1.61; 95% CI: 1.13, 2.30) and central adiposity (OR = 1.51; 95% CI: 1.07, 2.13) only in women (S1 Table). The interaction terms between parental and individual education were not significant for either sex (S1 Table).

Among men, being in the stable high compared to the stable low category of educational mobility was associated with lower odds of underweight (OR = 0.32; 95% CI: 0.12, 0.90) but higher odds of overweight (OR = 1.88; 95% CI: 1.11, 3.19). There was no significant association between educational mobility and obesity or central adiposity in men. Compared to the stable low category in women, all three other categories of educational mobility were associated with higher odds of obesity. Stable high (OR = 1.75; 95% CI: 1.03, 2.98) and upwardly mobile (OR = 1.60; 95% CI: 1.08, 2.37) categories in women were associated with central adiposity. Household wealth status was significantly associated with all categories of BMI and central adiposity in both sexes. In men, high compared to lowest household wealth status was significantly associated with lower odds of underweight but high odds of overweight, obesity and central adiposity in both model 1 and 2 Similar odds and associations were observed in women; however, no significant association was found for underweight. Separately for parental and individual education, and intergenerational educational mobility, household wealth was added to the full models (S1 Table). While only household wealth remained significantly associated with BMI and central adiposity in men, high parental education (OR = 1.83; 95% CI: 1.24, 2.70), stable high (OR = 1.90; 95% CI: 1.18, 3.08) and upwardly mobile (OR = 1.89; 95% CI: 1.04, 3.43) categories of educational mobility remained significantly associated with higher odds of obesity in women.

## Discussion and conclusion

This study sought to examine associations between key markers of current and lifetime SES and categories of BMI, as well as central adiposity in Ghanaian adults using the most recent data collected by WHO SAGE in Ghana. We found that in men, high parental education and stable high category of intergenerational education mobility were significantly associated with lower odds of underweight but with higher odds of overweight. In women, high compared to low parental and individual education; stable high and upward educational mobility compared to the stable low category were significantly associated with higher odds of obesity and central adiposity. High household wealth status was significantly associated with higher odds of overweight, obesity and central adiposity in both men and women. At the same time, wealthier households were significantly associated with lower odds of underweight making household wealth a protective factor against underweight especially in men. Finally, accounting for the post-stratified person weights, the prevalence of central adiposity was higher compared to the combined prevalence of overweight and obesity in both men and women, and the prevalence of overweight and obesity separately exceeded that of underweight in this population.

Significant findings made in this study were that the prevalence of central adiposity was high, and the prevalence of overweight and obesity separately exceeded underweight in both men and women. The later corroborates previous studies that showed that overweight/obesity exceeded underweight in some developing countries [17]. Historically, the prevalence of obesity in Ghana’s population was as low as 0.8% in 1980 [42, 43], it gradually increased to 5.5% in 2003 [44] and 9.8% in 2008 [24]. Our study shows a weighted obesity prevalence of 12.9% and unweighted obesity prevalence about 12% at the time of data collection (2014/2015). In 2003, the weight and height measures used to estimate BMI prevalence were self-reported (as part of SAGE Ghana Wave 0). Weight and height were then objectively measured in 2008 as part of SAGE Ghana Wave 1 (9.8% obese) impeding our ability to compare the prevalence rates in the two waves in 2003 and 2008 [45]. However, the current obesity rate estimated in this study also used objectively measured weight and height, making the current rate comparable to that of Wave 1 from 2008; and this shows an increase even in the case of unweighted prevalence. The increasing obesity rates could be due to several factors such as increased economic growth, rapid urbanization, changing food preference, and availability of healthy foods in Ghana over the past 15 years [46–48]. There is evidence of rapid urbanization in Ghana that has been accompanied by changes in dietary patterns, increase in sedentary lifestyles, increased commuting times due to traffic congestion hence commuters spend long sitting hours in traffic, the loss or lack of open and safe places for physical activities because these spaces are converted into residential or commercial building spaces, and less engagement in physical activities [46, 47]. Changes in dietary patterns could be associated with the readily available fast-foods as evidenced by almost a doubling of the hospitality sector (which includes restaurants and food services) from 2.9% to 5.5% from the year 2000 to 2010 in Ghana [47]. The combined effect of such conditions could serve as a platform for households to adopt lifestyles that make individuals prone to obesity as has been observed in other settings [17, 49]. However, as a major risk factor for non-communicable diseases, the high prevalence of obesity in this study compared to the historical prevalence emphasizes the need for deliberate actions to prevent obesity, a point which is supported by WHO’s position that an increasing prevalence of obesity may contribute to the rising burden of non-communicable diseases in Africa [1, 3, 16].

Another significant finding in this study is the result that especially in women, high compared to low parental and individual education levels were associated with higher odds of overweight and obesity. This finding is consistent with findings in most developing economies where overweight and obesity have been associated with high SES [13, 15, 50–52]. However, this finding is in contrast with results from countries such as Australia, Germany, the United States and Malaysia in which parental or an individual’s high educational attainment has been associated with low BMI, low central adiposity and good health outcomes [8, 10, 53–55]. The situation in Ghana suggests that high education which begins with the completion of SSS/SHS and above is an economic tool for resource acquisition through the generations. Hence, those whose parents had high education were likely to possess the resources needed to afford the ‘westernized’ lifestyle: increase consumption of fast foods, high sodium diets, and high caloric drinks which are associated with westernized diets and perceived to be major dietary changes that promote obesity [17, 56].

This phenomenon could also be explained by the potentially low level of awareness of the positive effect of maintaining healthy body weight especially among women and thus, suggests the need for nutritional education [57]. Furthermore, this may also be as a consequence of cultural influence, which portrays overweight and obesity instead of normal/healthy BMI as a sign of affluence and high social standing observed in some developing countries [5, 17, 57]. Our finding that high parental education is associated with lower odds of underweight in women supports the notion culture confers on body size and socio-economic status.

In this population and especially among women, having a stable high or upward educational mobility were significantly associated with higher odds of obesity (both defined by BMI and waist circumference). This finding, especially regarding central adiposity, confirms that high SES potentially affected obesity in Ghanaian women [15]. Our results, however, contradict findings in some previous studies conducted mostly in developed countries [8, 10, 11, 53]. For instance, Kuntz and Lampert found no significant increase in the risk of obesity among respondents with potentially upward educational mobility [10]; Gall et al found upward educational mobility was associated with higher healthy life scores that included low BMI in young Australians [8]; Albrecht and Gordon-Larsen reported upward education mobility was associated with low adult mean BMI [53]; Adina et al found high parental and high personal education levels were associated with lower odds of large waist circumference [54]. Thus, the association between educational mobility and obesity seem to differ based on the culture, social location, and economy from which a sample is drawn.

Evidence in this study that high household wealth increased the odds of overweight and obesity is consistent with findings from some developing economies [13, 17, 50]. For example, using educational attainment as an SES marker in 36 countries, Mendez et al [17] found a strong positive association between high SES and overweight/obesity in developing countries; but this association reduced or reversed for developed countries [50].

Another interesting finding in this study is that downward mobility in women compared to the stable low category was associated with higher odds of obesity, although such association was not seen with central adiposity. The sample of downwardly mobile populations overall was small, which is a positive result but may contribute to a relative underestimation of associations in this analysis. Given that, we have accounted for all possible observable factors, included the sampling weights, and used STATA’s GLLAMM multilevel multinomial logistic regression that adjusted for clustering and any latent variable, we could, therefore, rule out that this is not a spurious relationship. The finding is consistent with results in other studies in which positive associations were found between downward intergenerational education mobility and health risk outcomes [8, 10, 53].

Significantly low levels of education in women may have affected their food choices, preference, and understanding of the health implications of having unhealthy BMI or central adiposity. For many years to many children, SSS/SHS education was an “elusive dream” mainly due to the cost of obtaining this level of education and girls were mostly affected [31]. However, with increased general and nutrition education, a population is more likely to reduce such health inequalities [58], suggesting the need for increased general, health and nutrition education in the population. Alternatively, the results in this study suggest that education may be protective in reducing health inequalities in the population and thus, an improvement in education enrolment rates, especially in women, may reduce underweight in the population. Consequently, the overall results call for a balance in the implementation of management and preventive measures for all categories of BMI or waist circumference in the population. This notwithstanding, high prevalence of overweight/ obesity and the identified associations in this study coupled with the “obesogenic environment” created due to urbanization, makes it imperative that the public health system is well-resourced to prevent and manage, and further be ready to bear the burden overweight/ obesity may impose on the system.

This study has some limitations. First, the use of cross-sectional data prevented the determination of temporality, and therefore we were unable to discount the possibility of bias due to reverse causality. Second, the use of education to measure mobility for two different generations should be interpreted with caution since the applicability of education and education completion rates may differ in different generations. However, as the educational level is accurately recalled, it is more stable over time compared to other SES markers such as occupation; and the education gradient in health is considered robust when there are preventive and treatment methods for the health risk/outcome such as obesity [6–8]. Also, although the data is representative of the older adult population, analyses omit those observations with missing information such as height and weight, and this may have introduced selection bias which may affect internal validity even though the missing data were relatively minimal (less than 5%). Additionally, the small sample of younger adults aged 18–49 included in the study precludes strong conclusions about the relationship in younger adult ages. Finally, there are a few objective based measures we could not account for, like fast food diet, and occupation due to the scope of our paper and data limitations. Although we have respondents’ occupation in our data, it was not included for the following reasons: with regards to socio-economic factors that contribute to risk factors for health, education has shown to be the strongest and most consistent measure [6]; hence, our focus on education first and not occupation. Furthermore, the relationship between occupation and obesity are normally considered in the light of the income earned from such occupation, as well as, whether an individual’s occupation makes him/her active or sedentary. Although previous studies did not include factors such as physical activities [8, 15], we included both the level of physical activity determined using the Global Physical Activity Questionnaire (GPAQ) [36] which captures various activities including those at work, and household wealth which is a proxy for economic status of the household from which the individual originates. Thus, although we do not capture occupation in our model, its significance is captured by adjusting for physical activity and household wealth.

We recommend that future studies should adjust for measures such as individual genetic composition, availability of fast food diet, and household composition, which have the potential to influence obesity.

The study also has many strengths. First, we use waist circumference determined by the current SSA optimal cut-off which accounts for central adiposity hence obesity definition was not limited to only BMI. Secondly, we use the most current national dataset that is representative of a wide age group (≥18 years) in both sexes and the use of objectively measured anthropometric data that facilitates our ability to make inferences to the broader population. Finally, our ability to examine the relationship between intergenerational educational mobility and the different BMI categories and central adiposity fills a gap that has rarely been tested in LMICs.

In conclusion, our findings show that the prevalence of central adiposity was higher compared to the combined prevalence of overweight and obesity; and the prevalence of obesity was higher than that of underweight in the Ghanaian adult population. In contrast to studies from high-income countries, we found that high current and lifetime SES were associated with higher odds of overweight, obesity and central adiposity, and with lower odds of underweight in the population.

## Supporting information

S1 Table Sub-analyses between SES variables and BMI as well as central adiposity in Model 3. ‡ WC: Waist circumference.
(DOCX)

S1 File Strobe statement. (DOCX)
