# Supplementary material for: The role of intergenerational educational mobility and household wealth in adult obesity: Evidence from Wave 2 of the World Health Organization’s Study on global AGEing and adult health
Source: PLoS One. 2019 Jan 9;14(1):e0208491. doi: 10.1371/journal.pone.0208491 (PMC6326547; doi:10.1371/journal.pone.0208491)
Supplement: S1 Table — ‡ WC: Waist circumference. (DOCX) [file pone.0208491.s001.docx]

**Supplementary Table 1: Sub-analyses between SES variables and BMI as well as central adiposity in Model 3**

| **Men** | **Parental and Individual Education controlled simultaneously in Model 2** | | | | **Women** | **Parental and Individual Education controlled simultaneously in Model 2** | | | |
| --- | --- | --- | --- | --- | --- | --- | --- | --- | --- |
|  | **BMI**  **(**Ref: normal/healthy BMI) | | | **Central adiposity** (Ref: ^‡^WC <81.2cm) |  | **BMI**  **(**Ref: normal/healthy BMI) | | | **Central adiposity** (Ref: ^‡^ WC <80.0cm) |
|  | **Underweight** | **Overweight** | **Obese** | **WC ≥ 81.2** |  | **Underweight** | **Overweight** | **Obese** | **WC ≥ 80.0cm** |
|  | **OR [95% CI]** | **OR [95% CI]** | **OR [95% CI]** | **OR [95% CI]** |  | **OR [95% CI]** | **OR [95% CI]** | **OR [95% CI]** | **OR [95% CI]** |
| **Parental Education** | |  |  |  |  |  |  |  |  |
| Low | 1 | 1 | 1 | 1 |  | 1 | 1 | 1 | 1 |
| High | 0.42 (0.16, 1.08) | 1.51 (0.93, 2.44) | 1.89 (0.77, 4.65) | 1.06 (0.67, 1.63) |  | 0.35 (0.14, 0.89) * | 1.74 (1.20, 2.54) ** | 2.08 (1.37, 3.15) *** | 1.26 (0.86, 1.83) |
| **Individual Education** | |  |  |  |  |  |  |  |  |
| Low | 1 | 1 | 1 | 1 |  | 1 | 1 | 1 | 1 |
| High | 0.88 (0.58, 1.32) | 1.29 (0.99, 1.88) | 1.09 (0.54, 2.19) | 1.08 (0.81, 1.44) |  | 0.69 (0.38, 1.25) | 1.12 (0.81, 1.58) | 1.61 (1.13, 2.30) ** | 1.51 (1.07, 2.13) * |
|  | **Parental and individual Education interaction in Model 2** | | | |  | **Parental and individual Education interaction in Model 2** | | | |
| **Parental*Individual Education** | | |  |  |  |  |  |  |  |
| Low | 1 | 1 | 1 | 1 |  | 1 | 1 | 1 | 1 |
| High | 0.30 (0.04, 2.28) | 0.41 (0.09, 1.90) | 3.70 (0.39, 5.47) | 0.59 (0.57, 5.06) | | 0.68 (0.12, 3.90) | 0.83 (0.36, 1.93) | 0.76 (0.35, 1.64) | 0.76 (0.82, 2.50) |
|  | **SES variables controlled independently for household wealth in Model 2** | | | |  | **SES variables controlled for household wealth in Model 2** | | | |
| **Parental Education** | |  |  |  |  |  |  |  |  |
| Low | 1 | 1 | 1 | 1 |  | 1 | 1 | 1 | 1 |
| High | 0.47 (0.19, 1.18) | 1.43 (0.88, 2.31) | 1.62 (0.67, 3.94) | 0.98 (0.64, 1.49) | | 0.32 (0.13, 0.76) ** | 1.45 (1.02, 2.06) * | 1.83 (1.24, 2.71) ** | 1.36 (0.92, 2.00) |
| **Individual Education** | |  |  |  |  |  |  |  |  |
| Low | 1 | 1 | 1 | 1 |  | 1 | 1 | 1 | 1 |
| High | 0.98 (0.65, 1.49) | 1.08 (0.73, 1.60) | 0.82 (0.40, 1.67) | 0.92 (0.69, 1.23) | | 0.60 90.34, 1.07) | 1.01 (0.72, 1.41) | 1.35 (0.94, 1.93) | 1.46 (1.02, 2.08) * |
| **Intergenerational Education Mobility** | | |  |  |  |  |  |  |  |
| Stable Low | 1 | 1 | 1 | 1 |  | 1 | 1 | 1 | 1 |
| Stable High | 0.42 (0.15, 1.21) | 1.40 (0.79, 2.47) | 1.30 (0.48, 3.48) | 0.90 (0.55, 1.48) | | 0.22 (0.08, 0.66) ** | 1.31 (0.81, 2.11) | 1.90 (1.18, 3.08) ** | 1.56 (0.90, 2.71) |
| Upwardly | 1.40 (0.25, 4.09) | 3.04 (0.67, 6.78) | 0.28 (0.02, 3.16) | 1.53 (0.50, 4.69) | | 0.41 (0.12, 1.36) | 1.61 (0.93, 2.79) | 1.89 (1.04, 3.43) * | 1.33 (0.76, 2.33) |
| Downwardly | 1.13 (0.73, 1.73) | 1.07 (0.71, 1.61) | 0.66 (0.30, 1.44) | 0.94 90.70, 1.27) | | 0.75 (0.39, 1.42) | 0.92 (0.60, 1.42) | 1.17 (0.76, 1.80) | 1.46 (0.97, 2.19) |

^‡^ WC: Waist circumference
