# Supplementary material for: The role of intergenerational educational mobility and household wealth in adult obesity: Evidence from Wave 2 of the World Health Organization’s Study on global AGEing and adult health
Source: PLoS One. 2019 Jan 9;14(1):e0208491. doi: 10.1371/journal.pone.0208491 (PMC6326547; doi:10.1371/journal.pone.0208491)
Supplement: S1 File — (DOCX) [file pone.0208491.s002.docx]

Re: **PLOS ONE- PONE-D-18-11009 “Review: The Impact of Intergenerational Educational Mobility and Household Income on Adult Obesity: Evidence from WHO Study on Global Ageing and Adult Health**

**STROBE Statement—**Checklist of items that should be included in reports of cross-sectional studies

|  | **Item No** | **Recommendation** |
| --- | --- | --- |
| **Title and abstract** | 1 | 1. Indicate the study’s design with Title and abstract   **[Within the title page 1 and methods section of the abstract in page 3**] |
|  |  | 1. (b) Provide in the abstract an informative and balanced summary of what was done **[Find in results section of abstract in page 3]** |
| **Introduction** |  |  |
| Background | 2 | Explain the scientific background and rationale for the investigation being reported [**page 5 and 6**] |
| Objectives | 3 | State specific objectives, including any prespecified hypotheses **[See in page 6]** |
| **Methods** |  |  |
| Study design | 4 | Present key elements of study design early in the paper [**Methods page 7**] |
| Setting | 5 | Describe the setting, locations, and relevant dates, including periods of recruitment, exposure, follow-up, and data collection [**page 7**] |
| Participants | 6 | (a) Give the eligibility criteria, and the sources and methods of selection of participants [**page 7**] |
| Variables | 7 | Clearly define all outcomes, exposures, predictors, potential confounders, and effect modifiers. Give diagnostic criteria, if applicable [**page 8-11**] |
| Data sources/measurement | 8 | For each variable of interest, give sources of data and details of methods of assessment (measurement). Describe comparability of assessment methods if there is more than one group **[page 8-11**] |
| Bias | 9 | Describe any efforts to address potential sources of bias [**page 12**] |
| Study size | 10 | Explain how the study size was arrived at **[page 7**] |
| Quantitative variables | 11 | Explain how quantitative variables were handled in the analyses. If applicable, describe which groupings were chosen and why [**page 11-12**] |
| Statistical methods | 12 | (a) Describe all statistical methods, including those used to control for confounding **[page 11-12]** |
|  |  | (b) Describe any methods used to examine subgroups and interactions [ **page 11-12**] |
|  |  | (c) Explain how missing data were addressed [**page 7**] |
|  |  | (d) If applicable, describe analytical methods taking account of sampling strategy **[page 11-12**] |
|  |  | (e) Describe any sensitivity analyses [**NA**] |
| **Results** |  |  |
| Participants | 13 | (a) Report numbers of individuals at each stage of study—eg numbers potentially eligible, examined for eligibility, confirmed eligible, included in the study, completing follow-up, and analysed [**page 7**] |
|  |  | (b) Give reasons for non-participation at each stage [**NA**] |
|  |  | (c) Consider use of a flow diagram [**NA: Information provided in Tables 1, 3 and 4 provide summary**] |
| Descriptive data | 14 | (a) Give characteristics of study participants (eg demographic, clinical, social) and information on exposures and potential confounders [**Please see Results section in page 13 and Tables 1, 2, 3 and 4**.] |
|  |  | (b) Indicate number of participants with missing data for each variable of interest **[page 7]** |
| Outcome data | 15 | Report numbers of outcome events or summary measures [**page 13 and Tables 1-4**] |
| Main results | 16 | (a) Give unadjusted estimates and, if applicable, confounder-adjusted estimates and their precision (eg, 95% confidence interval). Make clear which confounders were adjusted for and why they were included [**Page 12, papagraph 2 and shown in Tables 3, 4, 5 and 6**] |
|  |  | (b) Report category boundaries when continuous variables were categorized [**NA**] |
|  |  | (c) If relevant, consider translating estimates of relative risk into absolute risk for a meaningful time period [**NA**] |
| Other analyses | 17 | Report other analyses done—eg analyses of subgroups and interactions, and sensitivity analyses [**Sub-analyses and interactions reported in pages 14 & 15**] |
| **Discussion** |  |  |
| Key results | 18 | Summarise key results with reference to study objectives [**page 16**] |
| Limitations | 19 | Discuss limitations of the study, taking into account sources of potential bias or imprecision. Discuss both direction and magnitude of any potential bias [**page 20**] |
| Interpretation | 20 | Give a cautious overall interpretation of results considering objectives, limitations, multiplicity of analyses, results from similar studies, and other relevant evidence [**page 16-20**] |
| Generalisability | 21 | Discuss the generalisability (external validity) of the study results [**page 20**] |
| **Other information** |  |  |
| Funding | 22 | Give the source of funding and the role of the funders for the present study and, if applicable, for the original study on which the present article is based [**page NA**] |
